# Controlled dehydration improves the diffraction quality of two RNA crystals

**DOI:** 10.1186/s12900-016-0069-1

**Published:** 2016-11-03

**Authors:** HaJeung Park, Tuan Tran, Jun Hyuck Lee, Hyun Park, Matthew D. Disney

**Affiliations:** 1 X-ray Core Facility, The Scripps Research Institute, Scripps Florida, 130 Scripps Way, Jupiter, FL 33458 USA; 2 Department of Chemistry, The Scripps Research Institute, Scripps Florida, 130 Scripps Way, Jupiter, FL 33458 USA; 3 Unit of Polar Genomics, Korea Polar Research Institute, Incheon, 21990 Republic of Korea; 4 Department of Polar Sciences, University of Science and Technology, Incheon, 21990 Republic of Korea

**Keywords:** Post-crystallization modification, RNA, Crystal dehydration, Free mounting system, Repeat expansion

## Abstract

**Background:**

Post-crystallization dehydration methods, applying either vapor diffusion or humidity control devices, have been widely used to improve the diffraction quality of protein crystals. Despite the fact that RNA crystals tend to diffract poorly, there is a dearth of reports on the application of dehydration methods to improve the diffraction quality of RNA crystals.

**Results:**

We use dehydration techniques with a Free Mounting System (FMS, a humidity control device) to recover the poor diffraction quality of RNA crystals. These approaches were applied to RNA constructs that model various RNA-mediated repeat expansion disorders.

**Conclusion:**

The method we describe herein could serve as a general tool to improve diffraction quality of RNA crystals to facilitate structure determinations.

**Electronic supplementary material:**

The online version of this article (doi:10.1186/s12900-016-0069-1) contains supplementary material, which is available to authorized users.

## Background

Even with sufficient size and volume, RNA molecules often yield poorly diffracting crystals for X-ray diffraction analysis. The nature of RNA, such as its repetitive negatively charged phosphate backbone, scarce functional groups, and high structural flexibility, results in poor packing of RNA molecules in crystals, which consequently leads to poor diffraction. For the same reasons, various crystal optimization efforts to improve diffraction quality often result in only a modest response. A common method to overcome this problem is to re-design the construct and repeat the crystallization screening until a perfect construct is obtained for diffraction experiments [1, 2].

Dehydration methods were utilized for many protein crystals, where the diffraction quality improved dramatically both in the resolution limit and mosaicity [3–5]. Crystal dehydration can be achieved by either vapor diffusion or humidity control instrumentations. In vapor diffusion, the main precipitants, used at a slightly higher concentration than used in the crystallization condition, are applied as the dehydration agents. The subject crystals are incubated for hours to days with a gradual increase of the dehydration agents [6]. A more rapid way of dehydration can be performed with humidity controlled devices such as Free Mounting System (FMS) and H1Cb, and an in-house made device [4, 5, 7]. The basic concept of the humidity controlled device is to encapsulate a bare crystal within a humidity- and temperature- controlled air stream and then gradually change the humidity to the desired level while observing the effect of dehydration by measuring the diffractions of the subject crystal. The advantage of the device over the vapor diffusion method is that the relative humidity (Rh) of a crystal can be fine-tuned over a desired time frame and the outcome of the dehydration is observed in real time. However, mounting a crystal onto a dehydration device requires some practice and only one crystal can be tested at a time.

Repeat expansion diseases are human genetic disorders affecting the nervous and muscular systems and are caused by the expansion of repeated microsatellite sequences in the coding or noncoding regions of the gene [8]. The repeat modules are generally three to six nucleotides in length [8, 9]. Crystal structures of these pathogenic repeat sequences could give insight into disease mechanisms and also give insights into the development of therapeutics [10]. Therefore, we have crystallized and determined structures of RNAs containing CCUG and AUUCU repeat sequences. The RNAs containing these repeats yielded a number of hits rather quickly in screening. However, none of the crystals diffracted beyond a ~15 Å resolution. We subjected these crystals to dehydration before designing and testing new constructs. Dehydration dramatically improved the diffraction limit of the crystals, and crystal structures were determined successfully and reported elsewhere [11, 12].

Although dehydration methods have been practiced to rescue many protein crystals of poor diffraction quality, there is only one report that describes this technique in detail for nucleic acid crystals in addition to an anecdotal account of *glmS* ribozyme crystals [13–15]. The dehydration approach used by Zhang and Ferre-D’Amare was to soak the poorly diffracting RNA crystals in a higher concentration of precipitants while exchanging cations to induce better contact among the RNAs in the crystal lattice. In *glmS* ribozyme crystals, the dehydration was unintentionally introduced by a stabilization solution for cryocooling. Our approach of crystal dehydration, however, was completed using FMS exclusively. The technique is also appropriate for the quick evaluation of RNA crystals with poor diffraction, even after they have grown to a sufficient size. Herein, we describe the dehydration method we have used to improve the diffraction quality of RNA crystals and our thoughts about the technique in general. This approach could have broad utility for structural studies of RNA crystals.

## Methods

### Crystallization screening

RNA samples containing three repeats of CCUG and two repeats of AUUCU were screened against the Nucleix Suite (Qiagen, Valencia, CA, USA) using a Gryphon crystallization robot (Art Robinsons, Sunnyvale, CA, USA) at room temperature. Crystals from hit conditions were tested for diffraction using the in-house X-ray diffraction system equipped with a Mar345dtb (Rayonix, Evanstone, IL, USA) and a Micromax 007 HFM (Rigaku Americas, The Woodlands, TX, USA). None of the tested crystals showed a promising diffraction pattern. Crystals obtained from precipitants containing 100 mM ammonium acetate, 5 mM MgSO_4_, 50 mM 2-(N-morpholino)ethanesulfonic acid, pH 6.0, and 600 mM NaCl were used to test our dehydration method.

### Dehydration protocol and diffraction imaging

Dehydration of the crystals was completed on the FMS (Rigaku Americas). The relative humidity (Rh) of the precipitant was determined to be ~96 %. To test the response of the crystals to dehydration, single crystals were mounted on a Litholoop (Molecular Dimensions, Altamonte Springs, FL, USA) and placed in a goniometer head. Diffraction images of the crystals were collected every 5 min, while the Rh of the crystals was reduced to 70 % at a gradient of 0.25 % Rh change per min. The best diffracting crystals with a Bragg spacing of 3.0 Å and 3.3 Å from the CCUG and AUUCU crystals, respectively, were harvested by following the established dehydration protocol. The crystals were coated with perfluoropolyether cryo oil (Hampton Research, Aliso Viejo, CA, USA) to prevent any change in humidity and were then immediately cryocooled by submersion in liquid nitrogen.

### Data collection and structure refinement

The diffraction dataset of a CCUG crystal in the space group of *P*4_1_2_1_2 was obtained on the PILATUS detector at beam line 11–1 of Stanford Synchrotron Radiation Lightsource, SLAC. The dataset of an AUUCU crystal in the space group of *P*4_1_ was obtained on the MAR-300 detector at beam line ID-G of LS-CAT, Advanced Photon Source, Argonne National Laboratory. Datasets were processed with iMOSFLM [16]. Structures were determined by molecular replacement using Phaser [17] with the tetraloop-tetraloop receptor of PDB ID 4FNJ [18] as the search model. The refined final structure was deposited under the PDB IDs 4 K27 and 5BTM for CCUG and AUUCU, respectively, and the research papers featuring the structures were published separately elsewhere [11, 12].

## Results and discussion

### Construct design and crystallization

**Fig. 1:**
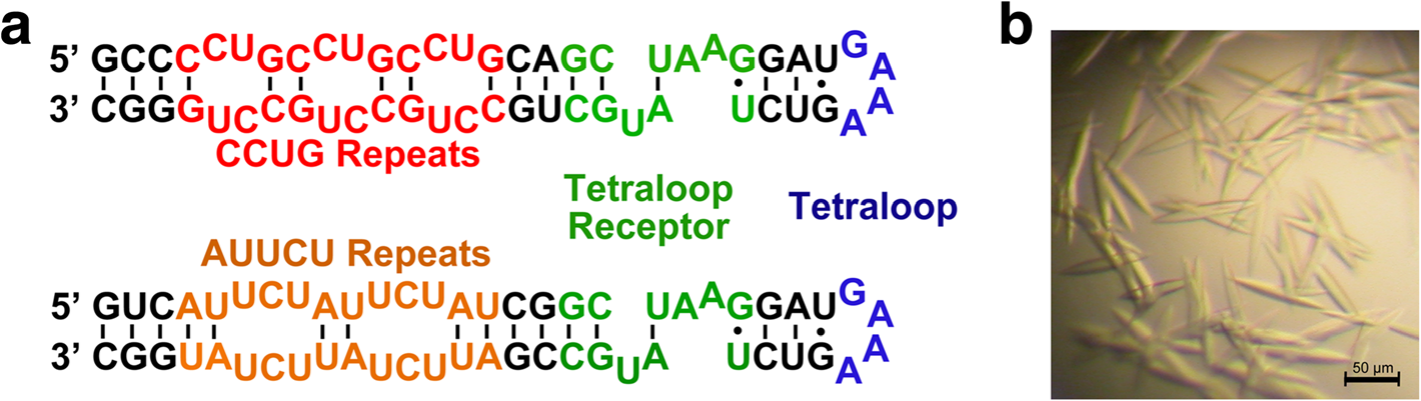
Design and crystallization of RNAs with repeat expansions. **a** Representations of the secondary structure of the RNAs used for crystallization and structure determination. **b** Picture showing the typical morphology of the crystals used for dehydration experiments

To overcome the inherent limitation of intermolecular crystal contact in RNAs, the GAAA tetraloop and the tetraloop receptor have been utilized as a general module to promote RNA crystallization [18, 19]. We applied this strategy and designed RNA constructs containing the target repeat sequences (Fig. 1a). Constructs were screened against the Nucleix suite (Qiagen), yielding a number of crystal hits with a tetragonal bipyramidal shape (Fig. 1b). However, none of the crystals tested showed a diffraction pattern. Before proceeding to redesign RNA sequences for new crystallization trials, we tested whether the non-diffracting crystals could be rescued by the dehydration technique.

### Overall structure and crystal packing

**Fig. 2:**
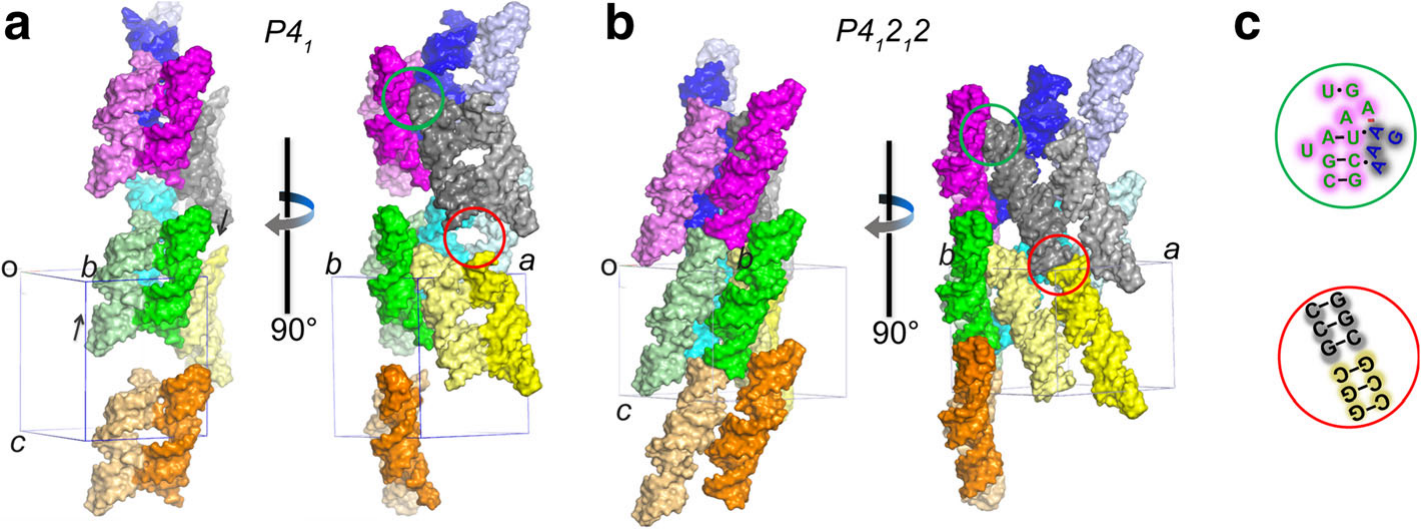
Comparison of the crystal packing between the AUUCU (**a**) and CCUG (**b**) crystals. The two asymmetric unit molecules of the AUUCU crystal are shown as pure and tinted colors in panel (**a**), and the equivalently arranged molecules in the CCUG crystal are shown with the same colors in panel (**b**). Tetraloop-tetraloop receptor interactions (e.g., green circled area of magenta and grey molecules in panels (**a**) and (**b**)) and the coaxial arrangement (e.g., red circled area of grey and yellow molecules in panels (**a**) and (**b**)) that appear invariant between the two crystals are shown as secondary structural drawings in panel (**c**). The two asymmetric molecules in the AUUCU crystal slid along the c-axis (*arrows*), making the interaction of the two molecules tighter than the equivalent interface in the CCUG crystal (e.g., *green* and *light green*)

The structure of the CCUG crystal showed that all bases were well ordered, with an overall B value of 35.1 Å^2^, and the electron density map correlated with the model. Detailed structural analysis can be found elsewhere [11]. On the contrary, approximately 20 % of the bases in the AUUCU crystal were not modeled owing to poor electron density. The affected residues were located at the stem end [12]. The most prominent crystal packing interactions of both crystal forms were mediated through tetraloop and tetraloop receptor interactions. Coaxial stacking and phosphate backbone interactions between neighboring RNA molecules were also observed in both crystals. Although the symmetry-related RNA molecules aligned coaxially in AUUCU crystals, base stacking between them could not be observed owing to disorder in the stem ends (Fig. 2).

Comparison of the molecular packing of the two crystal forms revealed that the overall molecular arrangements are very similar between the two crystals (Fig. 2). Distinct differences appeared to be caused by a slight rearrangement of two molecules in parallel; that is, the two asymmetric molecules in the AUUCU crystal and their equivalent molecules in the CCUG crystal. The asymmetric molecules slid along the c-axis compared with the equivalent molecules in the CCUG crystal, resulting in tighter interaction between the two asymmetric unit molecules, shorter unit cell values in the a- and b-axes, and the lower solvent content of the crystal. These observations also suggest the possibility that the space groups had diverged during the crystal transformation and the content of the RNA sequences could have dictated each space group formation. Space group transition during dehydration has been reported in monoclinic lysozyme crystals [20].

### Crystal dehydration and diffraction analyses

**Fig. 3:**
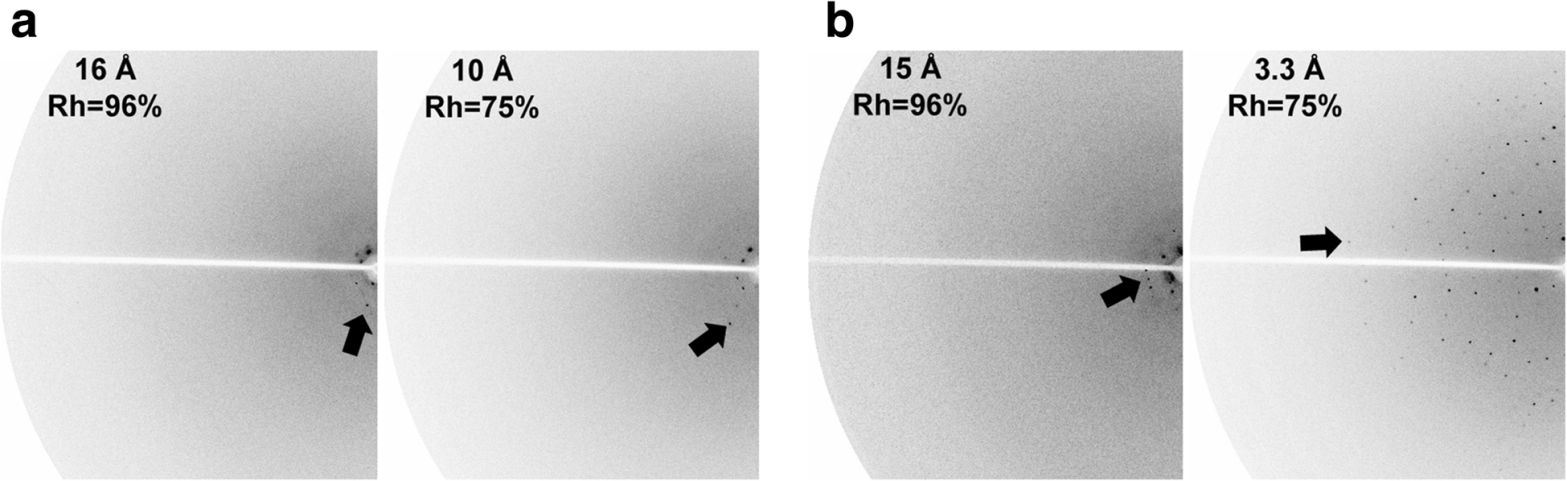
Improved diffraction limit in dehydrated crystals. In many cases, the tested crystals underwent a marginal improvement in the diffraction limit (**a**). Dramatic improvement of the diffraction limit permits structure determination (**b**)


Initial tests showed that the crystals responded to an Rh change through diffraction patterns in the 10–15 Å Bragg spacing range (Fig. 3a). Generally, these changes were too insignificant to affect the diffraction limits. Occasionally, however, crystals underwent a dramatic improvement in the diffraction quality (Fig. 3b). In such cases, the improvements in diffraction quality were noticeable at approximately 85 % Rh and improved continuously until 75 % Rh (Fig. 3 and Additional file 1: Movie). Lowering the Rh further reduced the resolution limit; thus, the crystals were prepared at 75 % Rh for synchrotron data collection.

Additional file 1. A series of diffraction images taken during the dehydration experiment is made as a movie file. (MP4 648 kb)


Diffraction analyses after the dehydration experiments revealed that the CCUG and AUUCU crystals were in different space groups in a primitive tetragonal lattice, even though the two crystals were morphologically identical and had emerged from the same precipitant. The CCUG crystal was in the *P*4_1_2_1_2 space group, with one molecule in the asymmetric unit. The unit cell values of the crystal were as follows: a = b = 75.08 Å, c = 59.90 Å, α = β = γ = 90°. The best crystal diffracted to 2.35 Å in a synchrotron radiation source. There was one molecule in the asymmetric unit with a Matthew’s coefficient value of 2.53 (51.37 % solvent content). The apparent space group of the AUUCU crystal was also point group 422 (*P*422). However, detailed analysis of the diffraction revealed that the crystal was merohedrally twined (twin fraction = 0.43). Therefore, the space group was lowered to point group 4. The crystal was also severely anisotropic. Molecular replacement followed by structural refinements confirmed the space group to be *P*4_1_. The final unit cell values of the crystal were a = b = 63.12 Å, c = 72.95 Å, and α = β = γ = 90°. The best crystal diffracted to 2.78 Å in a synchrotron radiation source. There were two molecules in the asymmetric unit with a Matthew’s coefficient value of 2.19 (43.75 % solvent content).

**Fig. 4:**
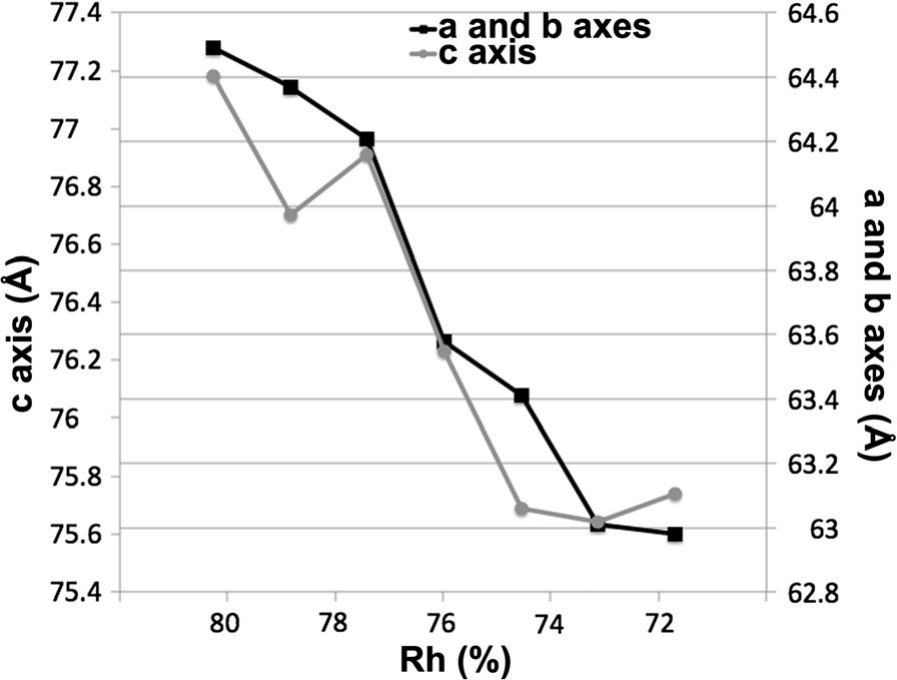
Relative humidity versus unit cell values during the dehydration experiment of an AUUCU crystal

Analysis of the individual diffraction images during dehydration of the AUUCU crystal (*P*4_1_) using HKL2000 [21] revealed that the dehydration process had introduced lattice shrinkage. The unit cell values calculated are shown in Fig. 4 and Table 1, along with the approximate humidity. Lattice shrinkage is a common observation in the dehydration process and has been reported by others [4, 22–24]. For example, dehydration of bovine mitochondrial F_1_-ATPase with orthorhombic crystals using FMS resulted in a more dramatic shrinkage of 12 and 6 % in the a- and c-axes, respectively, during the Rh change of 96 to 90 %. The lattice change in our crystal between Rh of 80 and 75 % was a 2.3 % reduction of the a- and b-axes and a 1.9 % reduction of the c-axis. The contractions are relatively minor compared to F_1_-ATPase even though Rh value decrease was about 15 %. Large contraction in protein crystals are related to domain motions and crystal contact improvement. Considering no diffraction in Rh range of 96 ~ 80 %, it is safe to assume that intermolecular contacts of RNAs in our crystals at this state are poor. The crystals are held together through tetraloop-tetraloop receptor interactions and possibly through unorganized phosphate backbone interactions of neighboring helices with coarse co-axial and parallel packing. The small changes in contractions beyond the Rh 80 % indicate the overall arrangements of the RNA molecules in the untreated crystals may not be much different than that in the dehydrated crystals. Also, the small changes of contractions after the dehydration unlikely influenced the RNA structures. Helical parameters of the RNAs from the two dehydrated crystals are comparable to those of RNAs without dehydration [11, 12]. The variation in the degree of shrinkage can be explained by variables such as crystal packing interactions, the flexibility of subject molecules (and subdomain movements), the size of the unit cell, and the solvent content.

**Table 1:** Effect of dehydration on unit cell values and diffraction limit of the AUUCU crystal (*P*4_1_)

Image No.	Rh (%)	a or b axis (Å)	c axis (Å)	Denzo distortion index (%)	Diffraction limit (Å)	Solvent content (%)
1 ~ 11	96 - 82	N/A	N/A	N/A	16 – 5.5	N/A
12	80	64.49	77.18	0.28	4	49.07
13	79	64.37	76.7	0.17	3.5	48.56
14	77	64.21	76.91	0.18	3.3	48.44
15	76	63.58	76.23	0.15	3	46.94
16	75	63.41	75.69	0.12	3	46.28
17	73	63.01	75.64	0.06	3.3	45.56
18	72	62.98	75.74	0.08	3.3	45.58
19	73	63.42	75.98	0.17	3	46.50
20	75	63.58	76.28	0.13	3	46.98

An earlier crystal dehydration study by Dobrianov et al., using tetragonal lysozyme, reported lattice shrinkage featuring nonlinear contraction of the crystal lattice and reproducible lattice transition points, beyond which irreversibility occurred (Rh value of 88 % for tetragonal lysozyme crystals) [23]. Such nonlinear contraction was also observed during the dehydration experiments of the bovine mitochondrial F_1_-ATPase [24]. Although inconclusive as a result of coarse data points, our RNA crystal also showed a similar trend, where the lattice shrinkage rate decreased towards the end of the dehydration experiment (Fig. 4 and Table 1).

### Points to consider for dehydration experiments

Although the response to dehydration was evident, it did not always guarantee high-quality diffraction at the end of the dehydration experiment. Within the same batch of crystals, there were crystal-to-crystal variations, where certain crystal diffracted well and others did not (Fig. 3). Visual inspection of the crystal could not identify or predict whether the crystal would yield quality diffraction after dehydration. Therefore, the identification of well-diffracting crystals was solely dependent on screening through dehydration experiments. Only about 13 %, or 2 out of 15 for CCUG crystals and 1 out of 8 AUUCU crystals, were of good diffraction quality. Bowler et al. also observed variability between crystals and their reaction to dehydration [24]. Therefore, if the diffraction quality of a tested crystal did not improve past the transition point (~80 % in our case), then the testing was terminated, and we moved on to a new crystal to save screening time. It needs to be further investigated whether the low reproducibility could be improved by modifying Rh gradient rate or other dehydration schemes.

Reported Rh value of saturated NaCl at 20 °C is 75 % [25]. Therefore, we also tested vapor diffusion dehydration following the methods published by Heras and Martin [6], where the concentration of reservoir NaCl, the main precipitant, was increase gradually from 600 mM to saturation point over a 5 day period. However, treatment had no effect on improving poor diffraction quality. The negative result could be because of an insufficient sample number (10 crystals were tested for diffraction), as we did not pursue the method extensively. Furthermore, the process of crystal dehydration using a higher concentration of precipitant takes longer to complete, as the sealed crystallization chamber needs to be equilibrated over hours to days, whereas dehydration by the FMS takes <2 h to complete. On the other hand, vapor diffusion dehydration can test multiple crystals with multiple variables simultaneously; controlled dehydration using the FMS must be completed serially.

## Conclusion

RNA molecules with multiple motifs are in general less likely to produce well-ordered crystals owing to the inherent flexibility of their structures and therefore require significant effort in the design and screening of constructs. The dehydration method presented here can be used as a routine technique to test RNA crystals with poor diffraction quality as an alternative to new construct screenings, provided that suitable instrumentation is available.

## Abbreviation

FMS: Free mounting system
